# Association and predictive value of the visceral adiposity index in diabetic kidney disease: a systematic review and meta-analysis

**DOI:** 10.3389/fendo.2026.1850744

**Published:** 2026-09-09

**Authors:** Jinying Chen, Weichen Nie, Ruixue Deng, Bo Dai, Jingsi Cao, Jieyu Zhang, Zheng Nan, Rui Chen

**Affiliations:** 1 The Affiliated Hospital of Changchun University of Chinese Medicine, Changchun, China; 2 Changchun University of Chinese Medicine, Changchun, China

**Keywords:** diabetic kidney disease, meta-analysis, predictive value, risk factor, systematic review, visceral obesity index

## Abstract

**Objective:**

The predictive value of the Visceral Adiposity Index (VAI) for diabetic kidney disease (DKD) is unclear. This study systematically reviews the association of elevated VAI with DKD, as well as its predictive performance.

**Methods:**

PubMed, Cochrane Library, Embase, Web of Science, CNKI, Wanfang, VIP, and CBM were searched until December 1, 2025, for studies investigating VAI and DKD. The pooled outcomes and effect measures included odds ratio (OR), hazard ratio (HR), area under the curve (AUC), sensitivity (SEN), specificity (SPEC), and diagnostic 2×2 tables. Additional analyses encompassed sensitivity analysis, heterogeneity assessment, and publication bias evaluation. Statistical analyses were enabled by Stata MP 15.1.

**Results:**

Nineteen studies on 58,312 participants were encompassed. Meta-analysis demonstrated that rising VAI was significantly associated with an elevated DKD risk (OR = 1.23, 95% CI: 1.14-1.33; HR = 1.12, 95% CI: 1.09-1.16). The pooled diagnostic performance of VAI for predicting DKD showed an AUC of 0.74 (95% CI: 0.70-0.77), with a SEN of 0.64 (95% CI: 0.55-0.73) and a SPEC of 0.72 (95% CI: 0.63-0.79).

**Conclusions:**

An increased VAI is associated with an elevated DKD risk and serves as an independent predictor. However, its predictive accuracy remains moderate. It cannot yet replace existing standards (such as urinary albumin-to-creatinine ratio (UACR) and the estimated glomerular filtration rate (eGFR)), but may serve as a complementary tool in resource-limited settings. Future research should explore whether combining VAI with other relevant indicators enhances the prediction of DKD risk.

**Systematic review registration:**

https://www.crd.york.ac.uk/PROSPERO/, identifier CRD42024587271.

## 1 Introduction

Among the most prevalent and serious microvascular complications of diabetes, diabetic kidney disease (DKD) is a primary cause of end-stage renal disease (ESRD) and places a heavy burden on global public health systems (1–3). Epidemiological studies have indicated that approximately 20%-40% of patients with diabetes will eventually develop DKD (4). Importantly, early diagnosis and timely intervention can significantly delay disease progression (5). The clinical diagnosis and risk stratification of DKD primarily rely on the urinary albumin-to-creatinine ratio (UACR) and the estimated glomerular filtration rate (eGFR) (6). Although these are cornerstone indicators, their limitations have been increasingly recognized. UACR is subject to variability, and a considerable proportion of patients present with non-albuminuric DKD, which may lead to underdiagnosis or delayed intervention (7, 8). Despite its widespread use, UACR remains suboptimal in terms of sensitivity (SEN), specificity (SPEC), and detection window (9, 10).

Traditional risk factors, including duration of diabetes, hyperglycemia, and hypertension, cannot fully explain the onset and progression of DKD, whereas obesity was an identified independent risk factor (11). Notably, accumulating evidence suggests that fat distribution is more pathophysiologically relevant than overall obesity indices such as body mass index (BMI) (12, 13). Therefore, identifying novel, reliable, and easily accessible biomarkers for risk prediction is of critical importance. Visceral adiposity has been closely linked to key drivers of DKD, including insulin resistance (IR), chronic inflammation, and endothelial dysfunction (14, 15). Visceral Adiposity Index (VAI) is a widely adopted indirect indicator of visceral adipose tissue function and distribution, reflecting not only fat quantity but also metabolic activity, and is considered more pathophysiologically informative than waist circumference (WC) or BMI. BMI reflects only overall body weight and cannot distinguish between muscle and fat mass. Moreover, it fails to account for the distribution of adipose tissue. Although WC can assess central obesity, it does not capture the metabolic disturbances associated with adipose tissue. Owing to its simplicity, accessibility, and good agreement with magnetic resonance imaging (MRI, gold standard), VAI is a recommended valuable surrogate marker for examining visceral fat (VF) accumulation and dysfunction (16). Increasing observational studies have explored VAI’s predictive value for DKD (17). Among the type 2 diabetes mellitus (T2DM) population, most cross-sectional and cohort studies demonstrated the significant relation of higher VAI to elevated proteinuria risk and a decline in eGFR (18, 19). However, inconsistencies in effect size (ES) between studies suggest that the predictive accuracy of VAI for DKD remains to be further clarified and validated.

In summary, the risk prediction of DKD remains challenging. As a simple, low-cost, and integrative indicator reflecting visceral adipose dysfunction, VAI shows considerable promise as an adjunctive predictive tool for DKD. Nevertheless, current evidence regarding the accuracy of VAI in predicting DKD is still insufficient. To date, no study has systematically evaluated the prospective predictive value of VAI (hazard ratio (HR)) alongside its diagnostic performance (SEN, SPEC, and area under the curve (AUC)). A previous meta-analysis evaluated the value of VAI in chronic kidney disease (CKD). However, it was limited to diagnostic accuracy metrics in a broad CKD population (20), whereas the present study specifically focused on DKD and further integrated prospective risk estimates (HRs). Therefore, the clinical utility of VAI as a screening tool for DKD remains to be fully elucidated. Our study expands the inclusion of studies presenting odds ratios (ORs) and first unravels HRs and diagnostic accuracy metrics to comprehensively assess the clinical value of VAI as a predictive tool for DKD.

## 2 Materials and methods

Our study followed the Preferred Reporting Items for Systematic Reviews and Meta-Analyses (PRISMA 2022) guidelines (21) and was registered in PROSPERO (ID: CRD42024587271) before commencement to ensure transparency and reproducibility. The checklist is shown in **Supplementary Material S1**.

### 2.1 Data sources and search strategy

PubMed, Cochrane Library, Embase, Web of Science, CNKI, Wanfang, VIP, and SinoMed databases were retrieved for eligible studies until December 1, 2025. The strategy involved Medical Subject Headings (MeSH) and free-text terms with core keywords “Diabetic Nephropathies” and “Visceral Adiposity Index”. No restrictions were imposed on region or publication year. The detailed search strategy is provided in **Supplementary Material 2**.

### 2.2 Eligibility criteria

Inclusion criteria were: (1) diabetic patients (≥18); (2) involved VAI measurement; (3) a comparator group (e.g., low VAI level group) was available; (4) at least one of the following outcomes could be extracted: association measures (OR, HR) or diagnostic performance metrics (AUC, SEN, SPEC); (5) observational design: cohort, case-control, or cross-sectional studies.

Exclusion criteria were: (1) although involving diabetic patients, the diagnosis of DKD was unclear or participants had concomitant diseases that could confound outcomes; (2) the exposure variable did not allow extraction of VAI-related indices; (3) relevant outcome data were unavailable or contained obvious errors; (4) the study type did not meet inclusion criteria, including animal studies, case reports, comments, conference abstracts, editorials, meta-analyses, or reviews.

### 2.3 Study selection

Studies were independently selected by two investigators with expertise in DKD (Jinying Chen and Ruixue Deng). After initial screening, cross-checking was conducted. Dissents were addressed by involving a third reviewer (Bo Dai). All retrieved records were uploaded to EndNote X9. Titles and abstracts were checked for removal of duplicates and irrelevant articles. Full texts of possibly eligible studies were subsequently screened. Those satisfying the eligibility criteria were ultimately analyzed.

### 2.4 Data extraction

Data were independently extracted by Jinying Chen and Bo Dai, with verification by a third reviewer (Ruixue Deng). A standard Excel was developed before the collection. Extracted information encompassed: study ID, title, first author, publication year, PMID/DOI, design (cohort, case-control, or cross-sectional), follow-up duration, country, data source, population characteristics, outcome (DKD), number of events, total sample size, sex distribution (male/female), age, threshold values, maximally adjusted OR or HR with 95% confidence intervals (CIs), and covariates encompassed in adjusted models. Participants were mainly from communities, hospitals, health examination centers, and public research databases. Multivariable regression models were used in the encompassed studies, adjusting for possible confounders: age, alanine aminotransferase (ALT), aspartate aminotransferase (AST), blood urea nitrogen (BUN), BMI, diastolic blood pressure, diabetes duration, drinking, education, eGFR, glycated hemoglobin (HbA1c), HOMA-IR, hypertension, income, lipid-lowering therapy, low-density lipoprotein cholesterol (LDL-C), medication use, race, serum creatinine, serum uric acid (SUA), sex, smoking, systolic blood pressure, total cholesterol (TC), and WC. Regarding association outcomes, results from multivariable-adjusted models were preferentially extracted.

### 2.5 Explanation of relevant values

DKD was defined according to established clinical criteria, including an eGFR<60 mL/min/1.73 m^2^ and/or a UACR ≥ 30 mg/g.

The VAI was calculated using the following formula (WC in cm, BMI in kg/m^2^, and triglyceride (TG) and high-density lipoprotein cholesterol (HDL-C) in mmol/L):


$$\text{Males}=\left(\frac{\text{WC}}{39.68+(1.88\times \text{BMI})}\right)\times \left(\frac{\text{TGs}}{1.03}\right)\times \left(\frac{1.31}{\text{HDL-C}}\right)$$

$$\text{Female}=\left(\frac{\text{WC}}{36.58+(1.89\times \text{BMI})}\right)\times \left(\frac{\text{TGs}}{0.81}\right)\times \left(\frac{1.52}{\text{HDL-C}}\right)$$

### 2.6 Study quality assessment

Case-control, cohort, and cross-sectional studies were analyzed. Therefore, different tools were applied by the study design for methodological quality and risk of bias (RoB) evaluation. The quality of case-control and cohort studies was rated via the Newcastle-Ottawa Scale (NOS), which examines study participant selection, comparability across groups, and exposure (for case-control studies) or outcome (for cohort studies) ascertainment, with 9 as the maximum score. ≥7, 4–6 and ≤3 signified high, moderate, and low quality. For cross-sectional ones, the 11-item Agency for Healthcare Research and Quality (AHRQ) checklist was employed, with every item scored 1 if met and 0 otherwise. 0-3, 4-7, and 8–11 denoted low, moderate, and high quality, respectively. For diagnostic accuracy studies, RoB was examined via QUADAS-2, which assesses patient selection, index test, reference standard, as well as flow and timing. Every domain was rated as “low”, “high”, or “unclear” RoB. RoB across encompassed studies was visualized via Review Manager (RevMan) 5.3. Two independent reviewers assessed the quality, with dissents addressed by a third reviewer.

### 2.7 Statistical analysis

Regarding association analyses, meta-analysis was carried out via Stata 15.1. To evaluate the association between VAI and DKD, ES was calculated as the natural logarithm of OR (ln OR), and the corresponding standard error (SE) was derived using the formula: SE=[ln (upper confidence limit) − ln (lower confidence limit)]/3.92. Heterogeneity among studies was assessed using Cochran’s Q test and quantified by the I^2^ statistic. Interpretation of I^2^ strictly followed the Seventh Cochrane Handbook for Systematic Reviews of Interventions, where 0%-40%, 30%-60%, 50%-90%, and 75%-100% indicate low or no, moderate, substantial, or considerable heterogeneity. A fixed-effects model (FEM) was utilized when I^2^<50%, whereas a random-effects model (REM) was employed when I^2^ ≥ 50%. Subgroup, sensitivity analyses, and meta-regression analyses were subsequently carried out for heterogeneity source confirmation.

For diagnostic and predictive analyses, meta-analysis was carried out via Stata 15.1 and Meta-Disc 1.4. Heterogeneity was examined utilizing Cochran’s Q test and the I^2^ statistic, and either FEMs or REMs were selected accordingly. Pooled estimates of SEN, SPEC, positive likelihood ratio (PLR), negative likelihood ratio (NLR), and diagnostic odds ratio (DOR), as well as 95% CIs, were derived. Forest plots and summary receiver operating characteristic (SROC) curves were generated, and AUC was computed. When heterogeneity was detected, subgroup analyses were carried out to explore its possible origins. Publication bias was examined via Deeks’ funnel plot, along with Egger’s and Begg’s tests, with two-sided P<0.05 denoting statistically significant bias. The influence of publication bias on the overall meta-analytic findings was estimated employing the trim-and-fill technique. Two-sided P<0.05 signified statistical significance.

## 3 Result

### 3.1 Study selection

**Figure 1:**
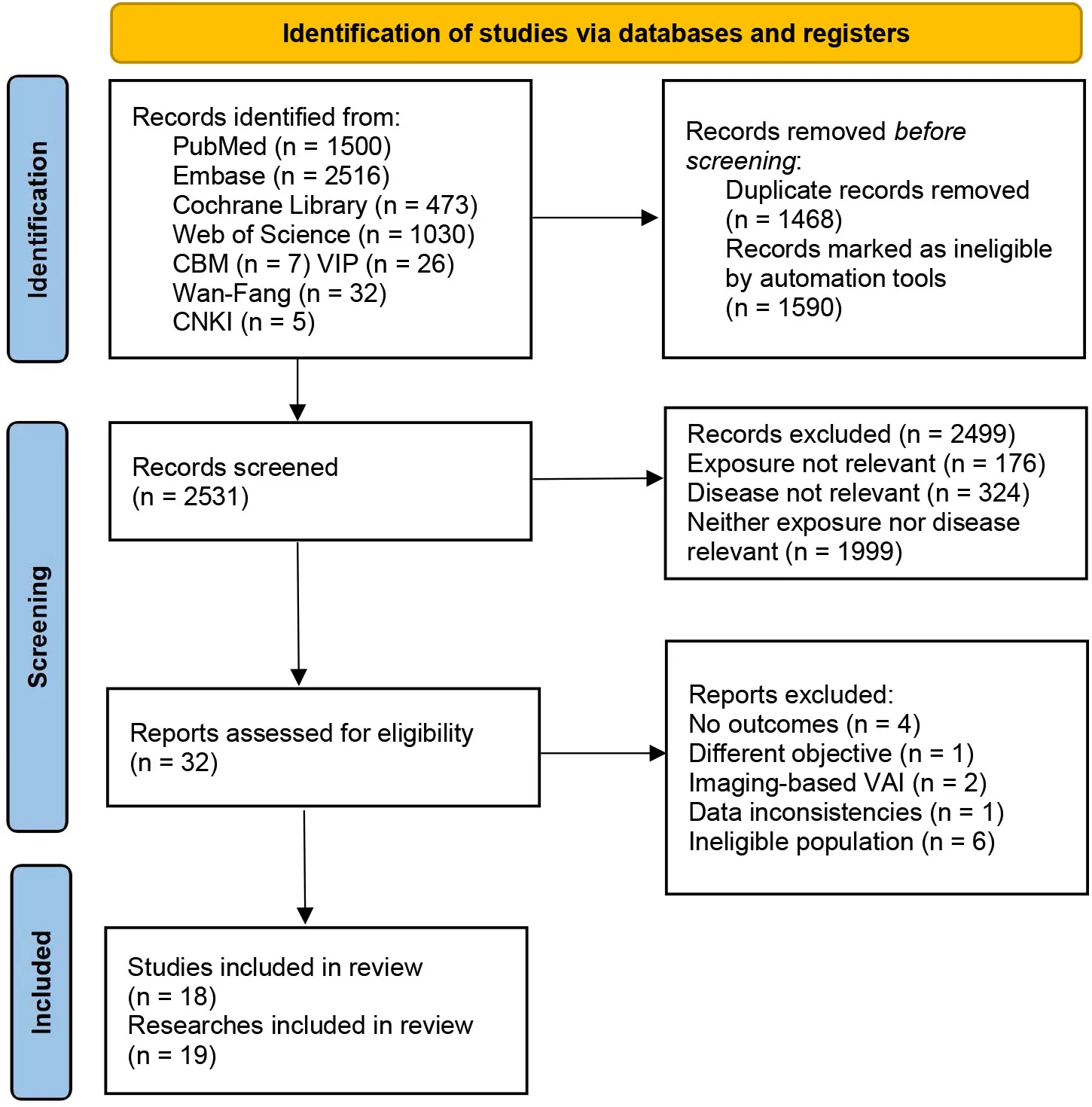
Flow diagram of study selection.

The literature screening process is shown in **Figure 1**. 5,589 records were initially identified up to December 1, 2025. After removing 1,468 duplicate records, 1,590 studies with inappropriate publication types were excluded using automated tools. 2,531 studies remained for initial screening. Based on title and abstract checking, 176 studies were excluded due to irrelevant exposure factors, 324 due to unrelated disease outcomes, and 1,999 due to both irrelevant exposure and disease, leaving 32 potentially eligible studies. During full-text review, 4 studies were excluded due to unavailable outcome data, 6 due to unclear DKD diagnosis or comorbid conditions, 1 due to differing outcome definitions, 2 due to VAI measurement based on imaging methods, and 1 due to obvious data errors. Ultimately, 18 articles comprising 19 studies were encompassed for meta-analysis.

### 3.2 Study characteristics

Characteristics of the encompassed studies are detailed in **Table 1**. Among the 19 studies, 5 were cohort studies (22–26), 13 were cross-sectional studies (18, 27–36), and 1 was a case-control study (37). There were 58,312 participants, including 3,156 from cohort studies, 240 from the case-control study, and 26,512 from cross-sectional studies. The follow-up for cohort studies was 4–14 years. Eligible studies were carried out in three countries, with 15 originating from China. Seventeen studies reported association outcomes, while ten studies provided data on predictive performance.

**Table 1:** Main characteristics of studies.

Study	Design	Region/Country	Mean age	Diagnostic methods	Sample size	Outcome Indicators
Zhiyuan Wu 2022 (22)	Cohort study	Beijing, China	53.35 (14.66)	UACR+eGFR	8948	HR, SEN, SPEC, AUC
Chun Zhou 2022 (23)	Cohort study	Nanfang Hospital, Southern Medical University, Guangzhou, Guangdong, China	62.8 ± 6.6	UACR+eGFR	10132	HR
Zhenzhen Sun 2022 (24)	Cohort study	Taiwan, China	55.91 ± 11.38	UACR+eGFR	2628	HR, SEN, SPEC, AUC
Zhenzhen Sun 2023 (25)	Cohort study	Taiwan, China	57.74 ± 11.42	UACR+eGFR	2659	HR, AUC
Ming Sun 2024 (26)	Cohort study	Tangshan, China	55.6 ± 10.0	UACR+eGFR	7193	HR
Feng He 2022 (37)	Case-control study	Gansu, China	56.99 ± 11.27	UACR+eGFR	240	OR, SEN, SPEC, AUC
Wen Jia 2020 (27)	Cross-sectional study	The Third Xiangya Hospital, Central South University, Changsha, Hunan, China	50.9 ± 8.8	UACR	341	OR
Licui Qi 2021 (18)	Cross-sectional study	Hebei, China	42.95 ± 11.57	UACR	335	OR, SEN, SPEC, AUC
Yu-Lun Ou 2021 (28)	Cross-sectional study	Taiwan, China	64.0± 11.3	UACR	1872	OR
Seyed Ali Nabipoorashraf 2023 (17)	Cross-sectional study	Tehran University of Medical Sciences, Tehran, Iran	56.46	UACR	2934	OR, SEN, SPEC, AUC
Chunyao Li 2024 (29)	Cross-sectional study	NHANES, United States	58.76 ± 0.36	UACR+eGFR	2508	OR
Pingping Zhao 2024 (30)	Cross-sectional study	The First Hospital of Lanzhou University, Lanzhou, Gansu, China	59.48 ± 11.03	UACR+eGFR	1241	OR
Wei Gu 2024 (31)	Cross-sectional study	Hebei, China	57.06 ± 12.53	UACR+eGFR	265	SEN, SPEC, AUC
Xin Zhao 2025 (32)	Cross-sectional study	Peking University First Hospital, Beijing, China	63.33 ± 8.71	UACR+eGFR	1817	OR, SEN, SPEC, AUC
Lu Zhang 2025 (33)	Cross-sectional study	NHANES, United States	57 ± 14	UACR+eGFR	2371	OR, SEN, SPEC, AUC
Fan Zhang a 2025 (34)	Cross-sectional study	Changzhou, China	51.0 (41.0, 61.0)	UACR+eGFR	1949	OR
Fan Zhang b 2025 (34)	Cross-sectional study	NHANES, United States	62.0 (51.0, 71.0)	UACR+eGFR	7158	OR
Qiuying Sun 2025 (35)	Cross-sectional study	Shandong, China	58.32 ± 22.23	UACR	1026	OR, SEN, SPEC, AUC
Mengdie Chen 2025 (36)	Cross-sectional study	The Second People’s Hospital of Yuhuan, Yuhuan, Zhejiang, China	70.0 (67.0, 73.0)	UACR+eGFR	2695	OR

UACR, urinary albumin/creatinine ratio; eGFR, estimated glomerular filtration rate; SEN, sensitivity; SPEC, specificity; OR, odds ratio; HR, Hazard Ratio.

### 3.3 Study quality assessment

According to NOS, all five cohort studies (22–26) had high quality, with scores of 7-8 (**Table 2**), primarily limited by insufficient follow-up duration or completeness. The single case-control study (37) was also rated as high quality with a score of 7 (**Table 3**), with minor limitations including suboptimal case definition and lack of reported response rates. For cross-sectional studies, quality was assessed using the AHRQ checklist. Four studies (18, 28, 30, 34) were rated as moderate quality (score=7) due to limitations such as unclear timeframes for patient identification, non-population-based or non-consecutive sampling, potential subjective bias in outcome assessment, lack of quality control descriptions, absence of missing data handling methods, and incomplete reporting of response rates and data integrity. Eight studies (17, 27, 29, 31–33, 35, 36) were of high quality, with scores of 8-10 (**Table 4**).

**Table 2:** NOS quality assessment of cohort studies.

Study	V1	V2	V3	V4	V5	V6	V7	V8	Overall
Zhenzhen Sun 2023 (25)	1	0	1	1	2	1	1	1	8
Zhiyuan Wu 2022 (22)	1	0	1	1	2	1	1	1	8
Zhenzhen Sun 2022 (24)	1	0	1	1	2	1	1	1	8
Chun Zhou 2022 (23)	1	1	1	1	2	1	0	0	7
Ming Sun 2024 (26)	1	1	1	1	1	1	1	1	8

V1, Representativeness of the exposed cohort; V2, Selection of the non-exposed cohort; V3, Ascertainment of exposure; V4, Demonstration that outcome of interest was not present at start of study; V5, Comparability of cohorts based on design or analysis; V6, Assessment of outcome; V7, Was follow-up long enough for outcomes to occur; V8, Adequacy of follow-up of cohorts.

**Table 3:** NOS quality assessment of case-control studies.

Study	V1	V2	V3	V4	V5	V6	V7	V8	Overall
Feng He 2022 (37)	0	1	1	1	2	1	1	0	7

V1, Whether the case definition is appropriate; V2, Representativeness of the cases; V3, Selection of controls; V4, Definition of controls; V5, Consideration of comparability between cases and controls in design and statistical analysis; V6, Ascertainment of exposure factors; V7, Use of the same method to ascertain exposure for cases and controls; V8, Non-response rate.

**Table 4:** AHRQ quality assessment of cross-sectional studies.

Study	D1	D2	D3	D4	D5	D6	D7	D8	D9	D10	D11	Overall
Chunyao Li 2024 (29)	Y	Y	Y	Y	Y	U	Y	Y	Y	U	NA	8
Licui Qi 2021 (18)	Y	Y	Y	U	Y	Y	Y	Y	U	N	NA	7
Lu Zhang 2025 (33)	Y	Y	Y	Y	Y	Y	Y	Y	U	U	NA	8
Pingping Zhao 2024 (30)	Y	Y	Y	U	Y	Y	Y	Y	U	N	NA	7
Seyed Ali Nabipoorashraf 2023 (17)	Y	Y	Y	Y	Y	Y	Y	Y	N	Y	NA	9
Wen Jia 2020 (27)	Y	Y	Y	Y	Y	Y	Y	Y	N	Y	NA	9
Yu-Lun Ou 2021 (28)	Y	Y	U	Y	N	Y	Y	Y	N	Y	NA	7
Wei Gu 2024 (31)	Y	Y	Y	U	Y	Y	Y	Y	N	Y	NA	8
Xin Zhao 2025 (32)	Y	Y	Y	Y	Y	Y	Y	Y	Y	Y	NA	10
Fan Zhang a 2025 (34)	Y	Y	Y	U	Y	U	Y	Y	Y	U	NA	7
Fan Zhang b 2025 (34)	Y	Y	Y	N	Y	U	Y	Y	Y	U	NA	7
Qiuying Sun 2025 (35)	Y	Y	Y	Y	Y	U	Y	Y	N	Y	NA	8
Mengdie Chen 2025 (36)	Y	Y	Y	Y	Y	U	Y	Y	Y	Y	NA	9

D1: Whether the source of information (e.g., survey, literature review) is clearly identified.

D2: Whether the inclusion and exclusion criteria for the exposed and non-exposed groups (cases and controls) are listed or reference is made to previous publications.

D3: Whether the time period for identifying patients is specified.

D4: If not population-based, whether the study subjects are consecutive.

D5: Whether subjective factors of the evaluators obscure other aspects of the study subjects.

D6: Whether any assessments conducted to ensure quality are described (e.g., testing/retesting of primary outcome indicators).

D7: Whether the reasons for patient exclusion are explained.

D8: Whether measures for evaluating and/or controlling confounding factors are described.

D9: If applicable, whether the handling of missing data in the analysis is explained.

D10: Whether the patient response rate and completeness of data collection are summarized.

D11: If follow-up is involved, whether the expected percentage of incomplete data for patients or the follow-up results is identified. Y: Yes; N: No; U: Unclear; NA: Not Applicable.

**Figure 2:**
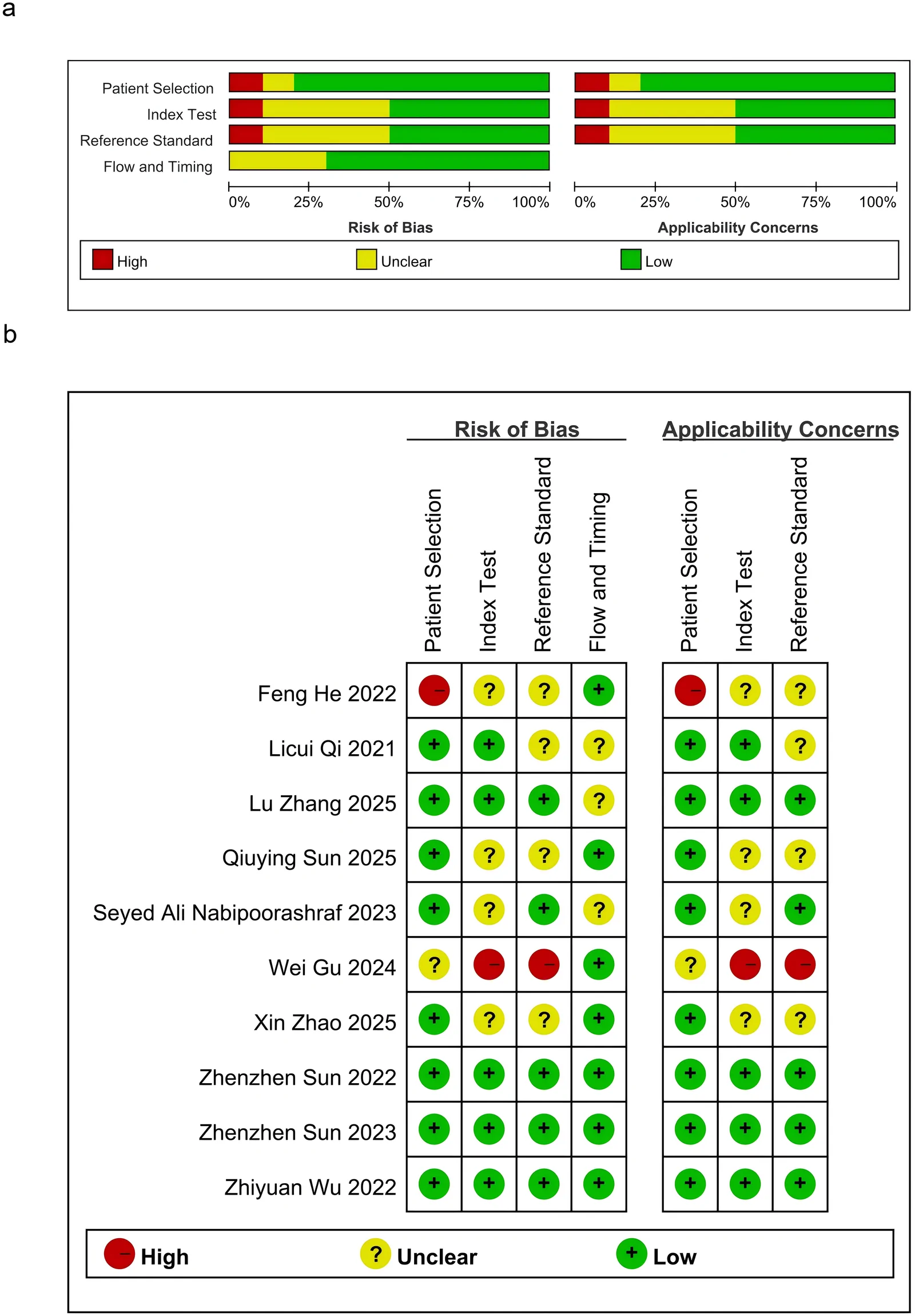
**(a)** QUADAS-2 methodological quality graph, **(b)** QUADAS-2 methodological quality summary.

Ten studies (17, 18, 22, 24, 25, 31–33, 35, 37) were encompassed in the diagnostic accuracy analysis and assessed employing QUADAS-2 (**Figure 2**). In patient selection, one study (37) exhibited a high risk owing to the case-control design, one study demonstrated unclear risk for uncertainty regarding consecutive or random sampling, and the remaining eight studies were of low risk. Regarding the index test, one study (31) had a high risk for no blinding to the reference standard and reported threshold. Four studies displayed unclear risk owing to uncertainty on pre-specified thresholds, while five studies were of low risk. As for the reference standard, one study (31) was judged as having a high risk because the reference standard interpretation was not blinded. Four studies were of unclear risk because of insufficient details on blinding procedures, and five studies were rated as displaying low risk. Concerning flow and timing, three studies exhibited unclear risk owing to a lack of clarity about whether an appropriate interval was maintained between the index test and reference standard; the remaining seven studies were of low risk.

### 3.4 Meta-analysis

#### 3.4.1 Correlation analysis

**Figure 3:**
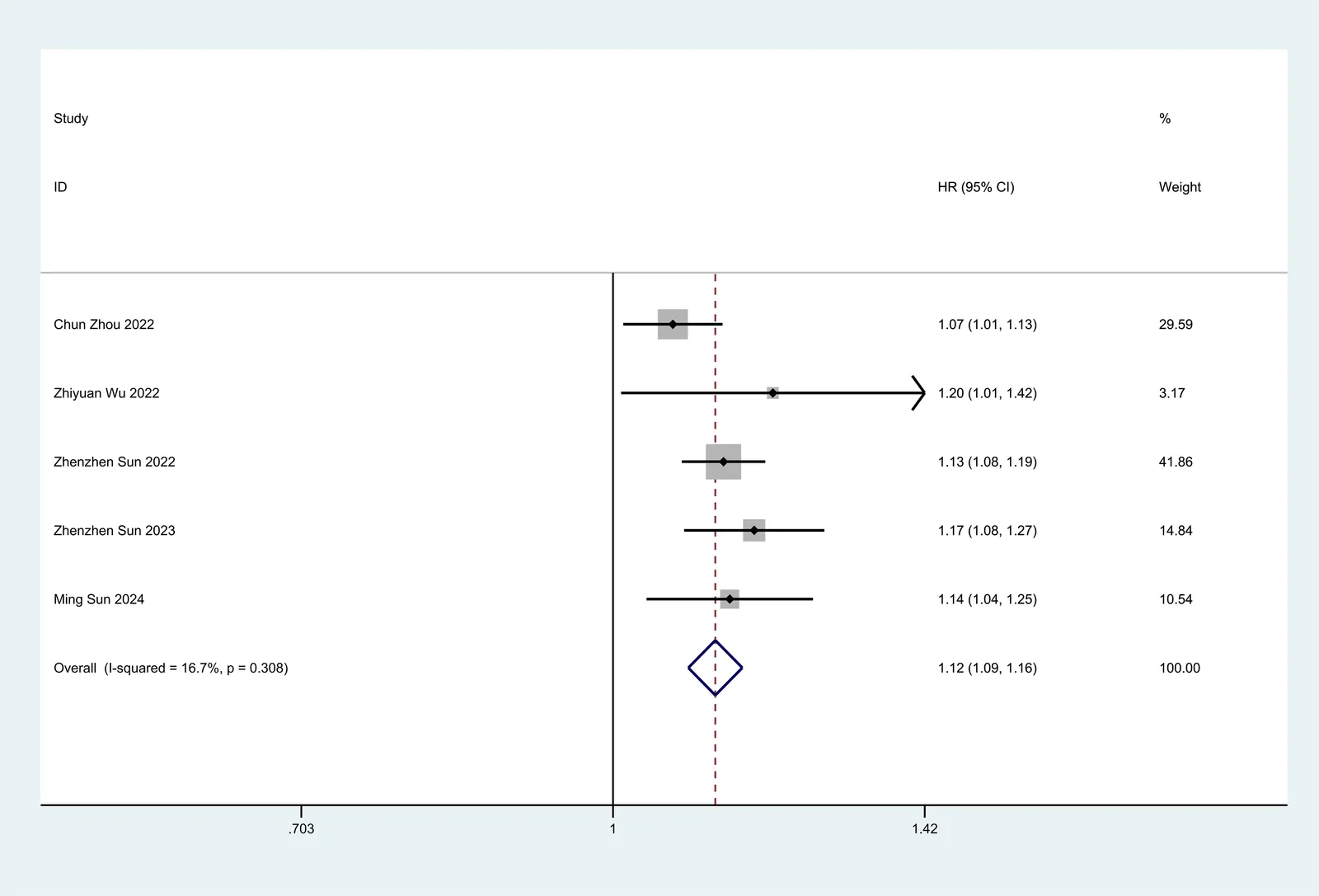
Forest plot for the correlation between VAI and DKD (HR).

Five studies (22–26) reported the correlation between VAI and the risk of diabetic nephropathy. The study results indicated that increased VAI was associated with a higher risk of diabetic nephropathy (HR = 1.12, 95% CI: 1.09-1.16). The effect test was statistically significant (z=7.42, P<0.001). The heterogeneity among studies was low (I^2^ = 16.7%, P = 0.308), so an FEM was used for analysis. The correlation results are shown in **Figure 3**.

**Figure 4:**
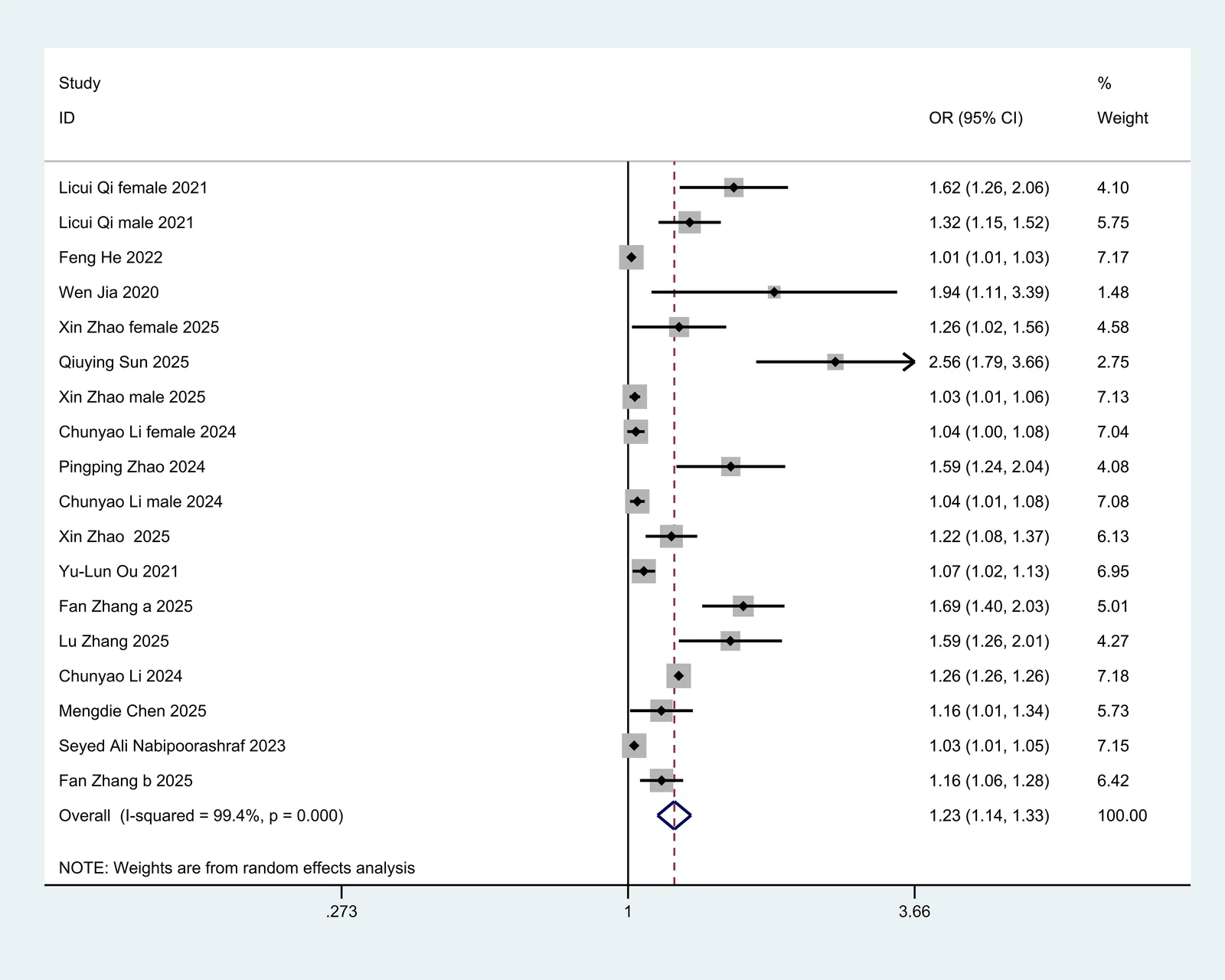
Forest plot for the correlation between VAI and DKD (OR).

Additionally, 12 articles comprising 18 studies (17, 18, 27–30, 32–37) evaluated the association between VAI and DKD risk. Due to substantial heterogeneity (I^2^ = 99.4%), an REM was used. Higher VAI was significantly associated with increased DKD risk (OR = 1.23, 95% CI: 1.14-1.33), with the overall effect being statistically significant (z=5.41, P<0.001) (**Figure 4**). To further compare sex differences, subgroup analyses stratified by sex were conducted. Given the substantial heterogeneity (I^2^ = 81.5%, P = 0.001), an REM was applied for pooling. The pooled OR was 1.26 (95% CI: 0.96-1.64) in females and 1.07 (95% CI: 1.01-1.14) in males. It should be noted that only three studies were included in each group of the above sex-stratified analyses, resulting in limited statistical power, wide CIs, and overlapping estimates. The association in the female subgroup did not reach statistical significance (OR = 1.26, 95% CI: 0.96-1.64). Therefore, no definitive conclusion can be drawn regarding sex differences (**Supplementary Figure 1**).

#### 3.4.2 Prediction accuracy analysis

**Figure 5:**
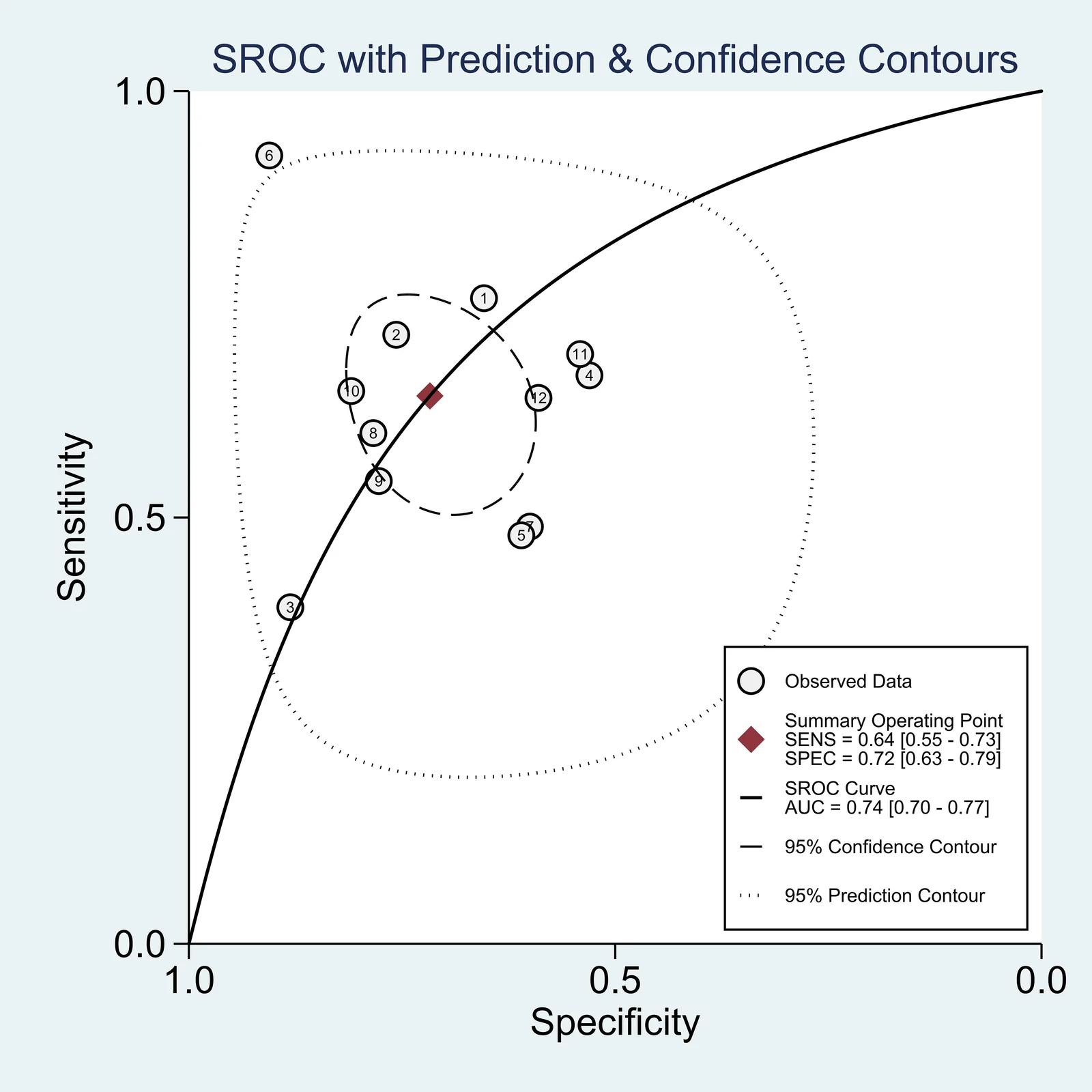
Summary receiver operating characteristic (SROC) curve of VAI for predicting DKD. AUC derived from diagnostic 2×2 table data.

**Figure 6:**
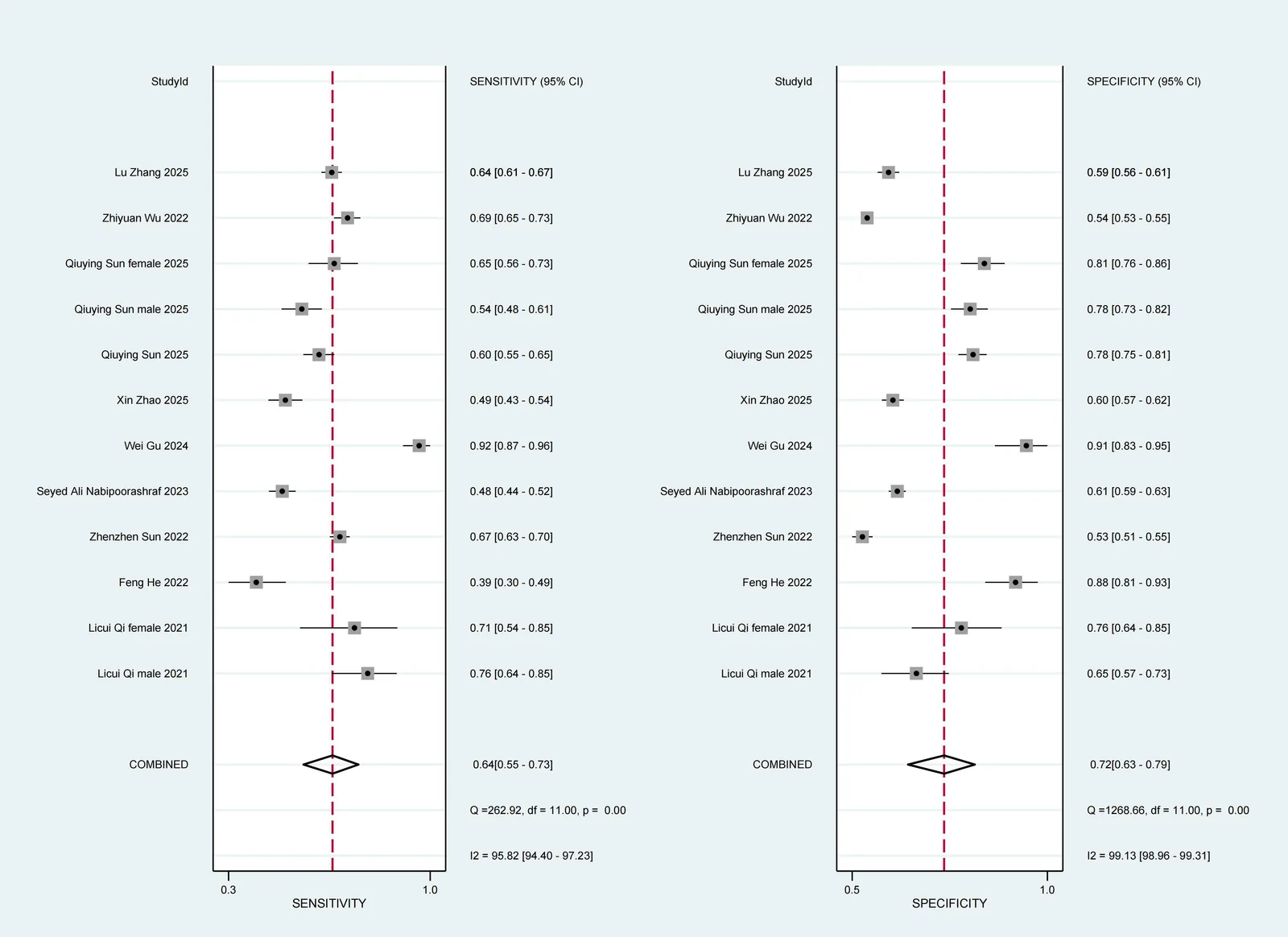
Forest plot for sensitivity and specificity.

Nine articles, including 12 studies (17, 18, 22, 24, 31–33, 35, 37), reported diagnostic 2×2 table data for predicting DKD using VAI. Using a REM, the pooled diagnostic performance was as follows: SEN: 0.64 (95% CI: 0.55-0.73; I^2^ = 95.82%, P<0.01), SPEC: 0.72 (95% CI: 0.63-0.79; I^2^ = 99.13%, P<0.01), PLR: 2.3 (95% CI: 1.6-3.2), NLR: 0.50 (95% CI: 0.37-0.67), and DOR: 5 (95% CI, 2-8). The SROC curve yielded an AUC of 0.74 (95% CI: 0.70-0.77) (**Figures 5**, **6**).

To further compare sex differences, subgroup analyses were conducted. In males, the pooled SEN was 0.59 (95% CI: 0.53-0.65; I^2^ = 90.70%, P = 0.001; **Supplementary Figure 2a**), and the pooled SPEC was 0.74 (95% CI: 0.70-0.78; I^2^ = 88.30%, P = 0.003; **Supplementary Figure 2b**). In females, the pooled SEN was 0.66 (95% CI: 0.59-0.73; I^2^ = 0.00%, P = 0.454; **Supplementary Figure 2c**), and the pooled SPEC was 0.80 (95% CI: 0.75-0.84; I^2^ = 0.00%, P = 0.328; **Supplementary Figure 2d**). In the aforementioned diagnostic sex-subgroup analyses, only two studies were included in each group, the CIs overlapped, and no formal statistical comparison between sexes was performed. The observed numerical differences should therefore be interpreted with caution, and no conclusion can be made regarding sex-related differences in diagnostic performance.

**Figure 7:**
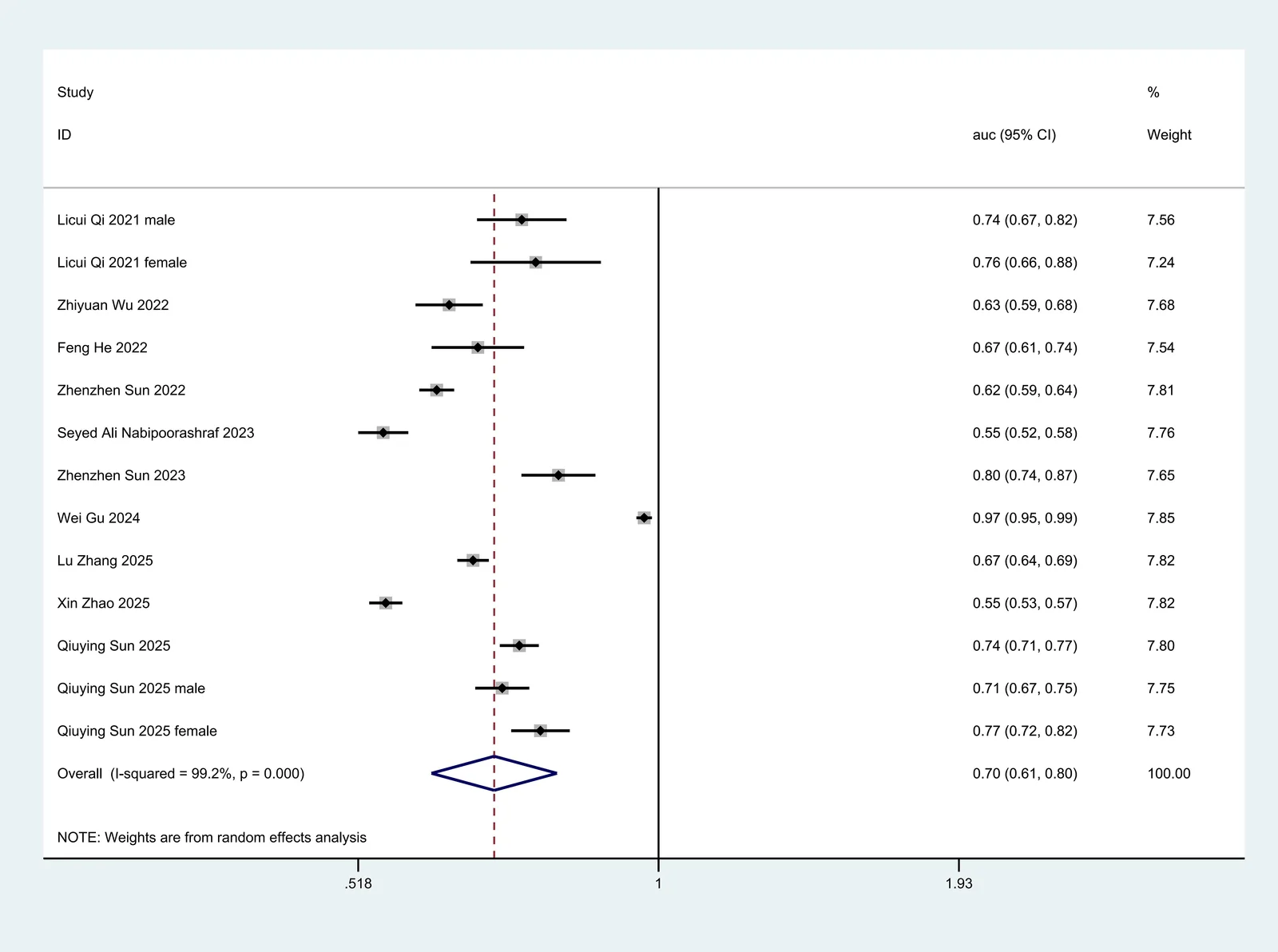
Forest plot of pooled AUC for diagnosing DKD. AUC values are directly reported by individual studies.

Furthermore, 10 articles, including 13 studies (17, 18, 22, 24, 25, 31–33, 35, 37), reported AUC values. The pooled analysis using an REM (I^2^ = 99.2%) showed that VAI had a moderate predictive value for DKD (AUC = 0.70, 95% CI: 0.61-0.80), which was notably greater than the null hypothesis (AUC = 0.5; z=5.13, P<0.001) (**Figure 7**). This value was obtained by directly pooling the AUCs reported in the original studies, whereas the AUC derived from the SROC curve based on diagnostic 2×2 table data in the first paragraph (0.74, **Figure 5**), which accounts for threshold effects and the correlation between SEN and SPEC, has been reported in the Abstract as the primary summary measure.

#### 3.4.3 Subgroup and meta-regression analysis

For the association between VAI and DKD (based on OR outcomes), subgroup analyses were conducted according to sex, study design, and diagnostic criteria, while meta-regression analyses by sample size and age were carried out. The analysis indicated that, in subgroup analyses, male sex, cross-sectional studies, cohort studies, UACR, and UACR combined with eGFR were sources of heterogeneity (**Table 5**). Meta-regression analysis further identified sample size as a significant contributor to heterogeneity (**Table 6**).

**Table 5:** Subgroup analysis for correlation studies.

Category	Research quantity	Effect size (OR)	*95%CI*	*I^2^*	*P*
Gender					
female	3	1.26	(0.96,1.64)	86.70%	0.091
male	3	1.07	(1.01,1.14)	82.70%	0.027
Design					
cross-sectional study	13	1.25	(1.14,1.36)	98.40%	0.000
cohort study	4	1.26	(1.03,1.54)	91.60%	0.024
Diagnostic methods					
UACR	7	1.30	(1.15,1.48)	98.80%	0.000
UACR+eGFR	11	1.12	(1.07,1.17)	87.50%	0.000

UACR, urinary albumin/creatinine ratio; eGFR, estimated glomerular filtration rate; OR, odds ratio.

**Table 6:** Correlation regression analysis.

Category	Coeff	Std.err	*t*	*P>| t |*	*95%CI*
sample size	0.2706	0.8367	3.23	0.005	(0.093,0.448)
age	1.4819	0.8683	1.71	0.119	(-0.453,3.417)

For the predictive performance of VAI (based on diagnostic 2×2 data), subgroup analyses by sex, study design, and diagnostic criteria were executed, while meta-regression analyses considered sample size and cut-off values. The analysis showed that, in subgroup analyses, male sex, cross-sectional studies, UACR, and UACR combined with eGFR were sources of heterogeneity in both SEN and SPEC (**Table 7**). Meta-regression analysis indicated that neither sample size nor cutoff value had a significant impact on predictive accuracy (DOR) (P > 0.05) (**Table 8**).

**Table 7:** Subgroup analysis in diagnostic test evaluation.

Category	Research quantity		Effect size		*95%CI*		*I^2^*		*P*	
	SEN	SPEC	SEN	SPEC	SEN	SPEC	SEN	SPEC	SEN	SPEC
Gender										
male	2	2	0.592	0.742	(0.535,0.648)	(0.704,0.778)	90.70%	88.30%	0.001	0.003
female	2	2	0.661	0.80	(0.587,0.730)	(0.75,0.84)	0.00%	0.00%	0.454	0.328
Design										
cross-sectional study	9	9	0.597	0.643	(0.579,0.616)	(0.631,0.654)	94.80%	96.20%	0.000	0.000
cohort study	2	2	0.676	0.539	(0.649,0.701)	(0.529,0.549)	0.00%	0.00%	0.354	0.401
Diagnostic methods										
UACR	6	6	0.560	0.673	(0.534,0.586)	(0.658,0.687)	86.30%	96.10%	0.000	0.000
UACR+eGFR	2	2	0.645	0.558	(0.627,0.663)	(0.549,0.566)	96.40%	96.80%	0.000	0.000

UACR, urinary albumin/creatinine ratio; eGFR, estimated glomerular filtration rate; SEN, sensitivity; SPEC, specificity.

**Table 8:** Diagnostic regression analysis.

Category	Coeff	Std.err	*P*	*DOR*	*95%CI*
sample size	0	0.0001	0.1261	1.00	(1.00, 1.00)
cut-off	0.006	0.0145	0.7142	1.01	(0.97,1.04)

#### 3.4.4 Sensitivity analysis and publication bias

For studies reporting association outcomes (HR), sensitivity analysis using an FEM indicated that no single study had a significant impact on the overall meta-analysis results (**Supplementary Figure 3a**). Funnel plot inspection showed approximate symmetry among the encompassed studies (**Supplementary Figure 3b**). Publication bias was further examined, and evidence of bias was insignificant (Begg’s test: z=0.49, P = 0.624 (**Supplementary Figure 3c**); Egger’s test: slope coefficient=0.074, P = 0.213 (**Supplementary Figure 3d**).

For studies reporting association outcomes (ORs), the funnel plot showed that the study estimates were distributed approximately symmetrically on both sides of the pooled ES, with most studies located near the top of the funnel plot, and no obvious asymmetry was observed (**Supplementary Figure 3e**). Begg’s test (z=3.45, P = 0.001) (**Supplementary Figure 3f**) and Egger’s test (slope coefficient=0.224, P<0.001) (**Supplementary Figure 3g**) both suggested the presence of publication bias. The results of Begg’s test after continuity correction were consistent (z=3.41, P = 0.001). In Egger’s test, the intercept for the bias term was −4.80 (P = 0.136), whereas the slope term was statistically significant.

For studies reporting predictive performance, sensitivity analysis similarly indicated that no individual study significantly influenced the pooled results (**Supplementary Figure 4a**). Deeks’ funnel plot also showed approximate symmetry (**Supplementary Figure 4b**). Although Deeks’ test suggested possible publication bias (P = 0.02), to evaluate its potential impact, a trim-and-fill analysis was performed using a linear estimator based on a FEM, imputing a total of five hypothetical studies. Given the substantial heterogeneity in the diagnostic accuracy analysis (I^2^ = 94.7%), the main results were reported using a REM, while the complete results from both fixed-effects and REMs are provided in the Supplementary Materials (**Supplementary Table 1**). Before trim-and-fill adjustment, the pooled lnDOR under the REM was 1.437 (95% CI: 1.077-1.797), P = 0.000. After adjustment, the pooled lnDOR under the REM was 2.301 (95% CI: 1.570-3.372), P = 0.000. The adjusted DOR was higher rather than lower than the original estimate, and both estimates remained statistically significant and consistent in direction. Subsequent trim-and-fill analysis indicated that this bias was unlikely to materially affect the overall results, suggesting that the findings remain relatively robust (**Supplementary Figure 4c**).

## 4 Discussion

This study systematically evaluated the association of VAI with both the risk and predictive value of DKD in the diabetic population. Based on 19 encompassing studies, a consistent and statistically significant positive association of VAI with DKD risk was observed, with minimal influence from publication bias, indicating that the findings are robust.

### 4.1 Gender differences and clinical significance

Our findings revealed certain sex-based differences in both risk association and predictive performance of VAI. However, only 2–3 studies were included in each subgroup analysis, resulting in limited statistical power, wide CIs, and overlapping estimates. Therefore, the observed numerical differences should be interpreted with caution, and no definitive conclusion can be drawn regarding the existence of true sex-related differences. The underlying mechanisms of these numerical differences may be related to sex hormone-mediated differential regulation of lipid metabolism and VF distribution.

At the physiological level, estrogen exhibits anti-inflammatory properties and enhances insulin sensitivity, thereby exerting protective effects on glucose and lipid metabolism in females. At physiological concentrations, estrogen suppresses inflammation via inhibition of the NF-κB pathway and improves insulin sensitivity by enhancing insulin signaling pathways. However, this protective effect is dose- and condition-dependent; it may be attenuated or even reversed under conditions of abnormally elevated estrogen levels (e.g., exogenous high doses) or in the presence of certain metabolic disorders such as polycystic ovary syndrome. Evidence suggests that, in premenopausal women, estrogen upregulates the expression of the long non-coding RNA Gas5 via the ERα receptor, thereby inhibiting IGF2BP1 to maintain homeostasis of visceral adipose tissue (38). Estrogen also improves insulin sensitivity, reduces abdominal fat accumulation, and maintains energy balance by modulating satiety-related gut peptides such as adiponectin and ghrelin (39). However, this protective effect reverses after menopause; with declining estrogen levels and alterations in the ERα/ERβ ratio, estrogen supplementation may instead exacerbate visceral adipose dysfunction and induce the production of pro-inflammatory cytokines and IR-related factors (38).

In males, androgen levels are significantly negatively correlated with VF accumulation. Longitudinal evidence indicates that declining testosterone levels are associated with increases in body weight, WC, and VF, along with reduced adiponectin levels (40). Epidemiological studies have demonstrated that males have significantly higher VF content compared to females (41). Moreover, VAI-related metabolic pathways, such as primary bile acid biosynthesis and branched-chain amino acid metabolism, exhibit sex-dependent differences (42).

The association of VAI with DKD was statistically significant in males but not in females, suggesting deeper metabolic heterogeneity underlying this sex disparity. However, the exact significance of this difference remains to be further validated. The higher baseline burden of VF in males, together with the close interplay among testosterone, VF, and adiponectin, enables VAI to more effectively capture cumulative metabolic risk in men. In contrast, the protective effects of estrogen in females may buffer the immediate metabolic impact of increased VF, but this protection is state-dependent and may reverse after menopause. Therefore, the performance of VAI as a risk marker for DKD may vary according to sex and reproductive status. These sex-stratified findings provide preliminary directions for future research, but given the limited number of studies included in the subgroup analyses, the conclusions require confirmation in larger prospective studies. Future studies should stratify analyses by menopausal status and explore sex-specific VAI thresholds.

### 4.2 Sources of heterogeneity and methodological considerations

This study also found that heterogeneity primarily arose from differences in study design and DKD diagnostic criteria. In the association analysis, cross-sectional studies exhibited substantial heterogeneity, whereas cohort studies showed relatively low heterogeneity, highlighting the impact of study design on the stability of effect estimates. Cross-sectional studies are more susceptible to reverse causality and unclear temporal relationships; although their pooled effects were directionally consistent with cohort studies, the latter should be considered the primary source of evidence (43).

In the diagnostic accuracy analysis, pooled SEN and SPEC indicated significant clinical heterogeneity. Subgroup analyses revealed that differences in DKD diagnostic criteria were a major source of heterogeneity. UACR primarily reflects glomerular filtration barrier damage, whereas eGFR reflects overall renal function decline; their distinct pathophysiological implications mean that combining them may obscure the differential predictive value of VAI across disease pathways (44). Nevertheless, most effect estimates across studies were closely clustered around the pooled ES, with only a few studies showing relatively lower estimates. Sensitivity analyses further confirmed that the overall conclusions remained stable.

Significant and largely unexplained heterogeneity was observed in the present meta-analysis. Although subgroup analyses and meta-regression were conducted according to factors such as study design, diagnostic criteria, and sex, most pooled I^2^ values remained well above 75% (e.g., I^2^ = 98.4% for pooled ORs and I^2^ = 96.4% for pooled SEN), and substantial residual heterogeneity persisted within subgroups. This suggests that differences in effect estimates across studies cannot be attributed solely to random error. The high I^2^ values observed in this study may partly reflect the large sample sizes of the included studies. I^2^ represents the proportion of total variation attributable to between-study heterogeneity, and in studies with large sample sizes, I^2^ may be systematically inflated even when the absolute magnitude of between-study differences is relatively small. Therefore, interpretation of I^2^ should consider the underlying sources of clinical heterogeneity rather than relying solely on its numerical value.

From a clinical perspective, substantial differences in population characteristics (e.g., baseline renal function and concomitant medications), DKD diagnostic criteria (UACR alone versus combined with eGFR), and VAI cut-off selection are likely important drivers of heterogeneity. From a methodological perspective, variations in study design (mixed cross-sectional and cohort studies) and inconsistency in confounder adjustment strategies may have further increased the dispersion of effect estimates. Such high heterogeneity limits the generalizability of the pooled ES and suggests that the predictive performance of VAI may be highly context-dependent. Future studies should adopt standardized definitions of DKD, implement more comprehensive confounding control, and explore the predictive value of VAI in more homogeneous populations.Furthermore, for diagnostic accuracy analyses, the traditional univariate I^2^ statistic has methodological limitations when evaluating paired SEN and SPEC because it does not account for the intrinsic trade-off between these two measures. The substantial variation in VAI cut-off values across studies suggests the presence of threshold effects, which represent a common and reasonable source of heterogeneity in diagnostic test meta-analyses. Under such circumstances, the overall diagnostic performance reflected by the SROC curve (AUC = 0.74) is more representative than pooled SEN or SPEC estimates alone, and the latter should be interpreted cautiously.

### 4.3 Comparison with previous studies

VAI outperforms conventional obesity indices in predicting metabolic complications. Although BMI is widely adopted for general obesity assessment, it cannot distinguish between lean mass and VF, and its reliability as a predictor of adiposity and metabolic risk has been increasingly questioned (45). Alkhalaqi et al. reported in a Qatari population that the predictive performance of VAI for T2DM is significantly superior to that of BMI, and the strength of the association between VAI and T2DM is greater than that observed for BMI (46). A meta-analysis by Fang et al. in patients with CKD also demonstrated favorable predictive performance of VAI, with SEN and SPEC estimates comparable to those observed in the present study, suggesting relatively consistent predictive capability across different populations and renal outcomes (47). Studies in Chinese populations further indicate that VAI integrates anthropometric and metabolic parameters, thereby overcoming the limitations of conventional indices (48). Notably, recent longitudinal evidence suggests that VF accumulation precedes the decline in eGFR (49). These findings support the utility of VAI in the risk screening of DKD, enabling the identification of high-risk individuals before significant renal function decline.

### 4.4 Predictive performance of VAI and its potential complementary role

The discriminative ability of VAI for DKD was moderate (AUC of the SROC curve=0.74; pooled original AUC = 0.70), and its SEN was below the ideal threshold generally expected for a stand-alone screening tool (typically≥0.80). A SEN of 0.64 indicates that approximately 36% of DKD cases would be missed if screening relied solely on VAI. Therefore, VAI should not be regarded as a substitute for established diagnostic standards such as UACR and eGFR, but rather as a complementary risk indicator.

Nevertheless, it is precisely this complementary role that may carry clinical value. In current clinical practice, DKD screening relies heavily on UACR testing. Although UACR is the standard approach, its accessibility is not universal, particularly in resource-limited primary healthcare settings. In such contexts, VAI may serve as an initial screening tool to identify individuals at highest risk who are most likely to benefit from prioritized UACR testing, thereby improving screening efficiency. Unlike imaging techniques (e.g., CT or MRI), VAI requires only routine lipid measurements and WC assessment, making it cost-effective and widely accessible, particularly in primary care settings or large-scale population screening (50).

Notably, the complementary nature of VAI should be emphasized. Even in resource-rich settings, a considerable proportion of patients with diabetes present with non-albuminuric DKD, a phenotype that may be overlooked by screening strategies relying solely on UACR. Elevated VAI reflects underlying metabolic dysfunction and lipotoxicity and may provide a risk signal that is complementary rather than redundant to UACR. This suggests that VAI and UACR may function not as substitutes but as complementary tools, and their combined use may offer a more comprehensive assessment of DKD risk. This hypothesis is consistent with our diagnostic meta-regression findings. Although substantial variation existed in the VAI cut-off values used across studies, diagnostic meta-regression indicated that cut-off value was not a significant source of heterogeneity in diagnostic performance (P = 0.714, **Table 8**). This finding suggests that the overall discriminative ability of VAI for DKD is relatively stable and not highly dependent on the selection of a single precise threshold, which may facilitate its implementation in clinical practice.

The clinical value of VAI lies not in its stand-alone accuracy but in its potential to enhance existing assessment strategies when standard approaches are unavailable or insufficient. Future studies should formally evaluate a stepwise screening strategy in which VAI is first used for risk stratification, followed by targeted UACR testing, to determine whether such an approach can improve cost-effectiveness and patient outcomes.

### 4.5 VAI and DKD mechanisms

The association between elevated VAI and DKD may be explained by several interrelated mechanisms. First, VAI reflects adipose tissue dysfunction and IR, which can induce glomerular hyperfiltration and mesangial expansion, ultimately leading to glomerulosclerosis (51, 52). Second, VAI is characterized by elevated triglycerides and reduced HDL-C, a lipid profile that promotes renal lipotoxicity. Ectopic lipid accumulation in podocytes and proximal tubular cells can trigger mitochondrial dysfunction, oxidative stress, and apoptosis (53–55)). Third, as a metabolically active tissue, visceral adipose tissue releases pro-inflammatory adipokines like IL-6 and TNF-α, which aggravate endothelial dysfunction and systemic inflammation, key contributors to proteinuria and renal fibrosis in individuals with diabetes (56, 57). Finally, visceral obesity is strongly linked to the activation of both the renin-angiotensin-aldosterone system and the sympathetic nervous system, which in turn promotes glomerular hypertension (58–60).

Therefore, VAI is not only a predictive marker but may also serve as an integrated biological indicator reflecting the impact of lipotoxicity on renal function. These findings provide a novel perspective for DKD prevention and management: beyond traditional glycemic and blood pressure control, targeting VF accumulation and its associated metabolic disturbances through lifestyle interventions, SGLT-2 inhibitors, or GLP-1 receptor agonists may be promising for preventing DKD.

### 4.6 Limitations

This study has several limitations. First, substantial heterogeneity was observed across studies in all major analyses. This heterogeneity may have arisen from multiple sources, including differences in VAI cut-off values, variations in DKD diagnostic criteria (i.e., inconsistent UACR thresholds and eGFR cut-off values), differences in diabetes type and disease duration among the included populations, as well as variations in study design and the extent of confounder adjustment. It should be noted that I^2^ may be systematically overestimated in the presence of large sample sizes; therefore, its interpretation should be considered in conjunction with the underlying sources of clinical heterogeneity rather than relying solely on its numerical magnitude. Second, with respect to diagnostic accuracy, the conventional univariate I^2^ statistic has limited applicability to paired SEN and SPEC estimates because it does not account for the inherent trade-off between these measures. The substantial variation in VAI cut-off values across studies suggests the presence of threshold effects, which represent a common and reasonable source of heterogeneity in meta-analyses of diagnostic accuracy studies. Under such circumstances, the overall diagnostic performance represented by the SROC curve is more informative than pooled point estimates of SEN or SPEC alone, and the latter should be interpreted with caution. Third, although the VAI formula incorporates sex-specific parameters, some subgroup analyses (e.g., sex-stratified analyses) included only a limited number of studies, which may have affected the robustness of the corresponding findings. Fourth, most included studies were conducted in Chinese populations, which may limit the generalizability of the findings to other ethnic groups. Fifth, differences across studies in DKD diagnostic criteria, VAI cut-off selection, and confounder adjustment strategies may have introduced measurement bias and residual confounding. Sixth, the literature search was restricted to the databases specified in the Methods section and did not include grey literature databases, clinical trial registries, or Google Scholar, which may have resulted in the omission of some unpublished studies. Finally, owing to data availability constraints, this meta-analysis was unable to incorporate prediction interval estimates or diagnostic sensitivity analyses based on influence analysis. Future studies should incorporate these methods when sufficient evidence becomes available to provide a more comprehensive assessment of the robustness of the pooled estimates and the expected distribution of the true effect.

## 5 Conclusion

This systematic review and meta-analysis demonstrated a consistent positive association between elevated VAI levels and the risk of DKD. Exploratory subgroup analyses suggested potential numerical differences in the strength of association and predictive performance between sexes; however, these findings were based on a limited number of studies and should be regarded as hypothesis-generating. VAI may serve as a complementary risk stratification marker for DKD in patients with diabetes, although its discriminatory performance is modest and its sensitivity is insufficient for use as a standalone screening tool. VAI cannot currently replace established diagnostic standards such as UACR or eGFR. However, it may have potential clinical utility as a complementary approach in resource-limited settings, and its combined use with other indicators warrants further investigation. The clinical utility of VAI requires validation in adequately powered prospective studies across diverse populations, and the necessity of sex-specific cut-off values remains to be explored.

## Data Availability

The original contributions presented in the study are included in the article/**Supplementary Material**. Further inquiries can be directed to the corresponding authors.
