# Supplementary material for: Association and predictive value of the visceral adiposity index in diabetic kidney disease: a systematic review and meta-analysis
Source: Front Endocrinol (Lausanne). 2026 Sep 9;17:1850744. doi: 10.3389/fendo.2026.1850744 (PMC13597435; doi:10.3389/fendo.2026.1850744)
Supplement: Supplementary file 2 [file Table2.docx]

**Supplementary Material S2. Database Retrieval Strategies**

| Pubmed | | |
| --- | --- | --- |
| **Search number** | **Query** | **Results** |
| #1 | "Diabetic Nephropathies"[Mesh] | 32,777 |
| #2 | diabetes nephropathy[Title/Abstract] OR Diabetic Glomerulosclerosis[Title/Abstract] OR Diabetic Kidney Diseases[Title/Abstract] OR diabetic nephropathy[Title/Abstract] OR diabetic nephrosis[Title/Abstract] OR diabet*[Title/Abstract] OR diabet* Kidney[Title/Abstract] OR diabet* nephro*[Title/Abstract] OR Glomerulosclerosis, Nodular[Title/Abstract] OR Intracapillary Glomerulosclerosis[Title/Abstract] OR Kidney Diseases, Diabetic[Title/Abstract] OR Kimmelstiel Wilson Disease[Title/Abstract] OR Kimmelstiel Wilson Syndrome[Title/Abstract] OR Nephropathies, Diabetic[Title/Abstract] OR Nephropathy, Diabetic[Title/Abstract] OR Nodular Glomerulosclerosis[Title/Abstract] OR renal diabetes[Title/Abstract] OR Syndrome, Kimmelstiel-Wilson[Title/Abstract] OR DKD[Title/Abstract] OR DN[Title/Abstract] | 913,844 |
| #3 | visceral adiposity index[Title/Abstract] OR visceral adipose index[Title/Abstract] OR visceral adiposity[Title/Abstract] OR obesity indices[Title/Abstract] OR Visceral Obesity Index[Title/Abstract] OR VOI[Title/Abstract] OR VAI[Title/Abstract] | 7,621 |
| #4 | #1 OR #2 | 916,619 |
| #5 | #3 AND #4 | 1,500 |

| Embase | | |
| --- | --- | --- |
| **Search number** | **Query** | **Results** |
| #1 | 'diabetic nephropathy'/exp | 65687 |
| #2 | 'diabetes nephropathy':ab,ti OR 'diabetic glomerulosclerosis':ab,ti OR 'diabetic kidney diseases':ab,ti OR 'diabetic nephropathy':ab,ti OR 'diabetic nephrosis':ab,ti OR diabet*:ab,ti OR 'diabet* kidney':ab,ti OR 'diabet* nephro*':ab,ti OR 'glomerulosclerosis, nodular':ab,ti OR 'intracapillary glomerulosclerosis':ab,ti OR 'kidney diseases, diabetic':ab,ti OR 'kimmelstiel wilson disease':ab,ti OR 'kimmelstiel wilson syndrome':ab,ti OR 'nephropathies, diabetic':ab,ti OR 'nephropathy, diabetic':ab,ti OR 'nodular glomerulosclerosis':ab,ti OR 'renal diabetes':ab,ti OR 'syndrome, kimmelstiel-wilson':ab,ti OR dkd:ab,ti OR dn:ab,ti | 1424938 |
| #3 | #1 OR #2 | 1434524 |
| #4 | 'visceral adiposity index'/exp | 1274 |
| #5 | 'visceral adiposity index':ab,ti OR 'visceral adipose index':ab,ti OR 'visceral adiposity':ab,ti OR 'obesity indices':ab,ti OR 'visceral obesity index':ab,ti OR voi:ab,ti OR vai:ab,ti | 12744 |
| #6 | #4 OR #5 | 12979 |
| #7 | #3 AND #6 | 2516 |

| Cochrane Library | | |
| --- | --- | --- |
| **Search number** | **Query** | **Results** |
| #1 | Diabetic Nephropathies | 2137 |
| #2 | (Diabetic Kidney Diseases OR Diabetic Nephropathy OR Diabetic Glomerulosclerosis OR Kimmelstiel Wilson Disease OR Nodular Glomerulosclerosis OR Kimmelstiel Wilson Syndrome OR Intracapillary Glomerulosclerosis OR diabetes nephropathy OR diabetic nephropathies OR diabetic renal disease OR diabetic nephrosis OR renal diabetes OR diabet* Kidney OR diabet* nephro* OR DKD OR DN):ti,ab,kw | 21295 |
| #3 | (visceral adiposity index OR visceral adipose index OR visceral adiposity OR obesity indices OR Visceral Obesity Index OR VAI):ti,ab,kw | 21522 |
| #4 | #1 OR #2 | 21306 |
| #5 | #3 AND #4 in Trials | 473 |

| Web of Science | | |
| --- | --- | --- |
| **Search number** | **Query** | **Results** |
| 1 | TS=(Diabetic Kidney Diseases OR Diabetic Nephropathy OR Diabetic Glomerulosclerosis OR Kimmelstiel Wilson Disease OR Nodular Glomerulosclerosis OR Kimmelstiel Wilson Syndrome OR Intracapillary Glomerulosclerosis OR diabetes nephropathy OR diabetic nephropathies OR diabetic renal disease) | 67298 |
| 2 | TS=(visceral adiposity index OR visceral adipose index OR visceral adiposity OR obesity indices OR Visceral Obesity Index OR VAI) | 171796 |
| 3 | #1 AND #2 | 1030 |

| China National Knowledge Infrastructure | |
| --- | --- |
| Query | Results |
| TKA=糖尿病肾病+糖尿病肾脏病+糖尿病肾脏疾病 AND TKA=内脏脂肪指数+内脏肥胖指数 | 5 |

| The China Science and Technology Journal Database | |
| --- | --- |
| Query | Results |
| (((((((((((((((题名或关键词=糖尿病肾病 OR 题名或关键词=diabetes nephropathy) OR 题名或关键词=diabetic nephropathy) OR 题名或关键词=diabetic nephrosis) OR 题名或关键词=renal diabetes) OR 题名或关键词=糖尿病性肾小球硬化症) OR 题名或关键词=糖尿病性肾病) OR ((((((摘要=糖尿病肾病 OR 摘要=diabetes nephropathy) OR 摘要=diabetic nephropathy) OR 摘要=diabetic nephrosis) OR 摘要=renal diabetes) OR 摘要=糖尿病性肾小球硬化症) OR 摘要=糖尿病性肾病)))) OR ((((题名或关键词=糖尿病肾脏疾病 OR 题名或关键词=diabetic kidney disease) OR 题名或关键词=糖尿病肾脏病变) OR 题名或关键词=糖尿病性肾脏疾病) OR 题名或关键词=糖尿病肾脏病)))) OR ((((摘要=糖尿病肾脏疾病 OR 摘要=diabetic kidney disease) OR 摘要=糖尿病肾脏病变) OR 摘要=糖尿病性肾脏疾病) OR 摘要=糖尿病肾脏病)))) AND 摘要=内脏脂肪指数 | 26 |

| Wanfang Data | |
| --- | --- |
| Query | Results |
| (题名或关键词:(糖尿病肾病) or 题名或关键词:(糖尿病肾脏病) or 题名或关键词:(糖尿病肾脏疾病) or 摘要:(糖尿病肾病) or 摘要:(糖尿病肾脏病) or 摘要:(糖尿病肾脏疾病)) and( 题名或关键词:(内脏脂肪指数) or 题名或关键词:(内脏肥胖指数) or 摘要:(内脏脂肪指数) or 摘要:(内脏肥胖指数) ) | 32 |

| China Biology Medicine disc | |
| --- | --- |
| Query | Results |
| ("内脏脂肪指数"[常用字段] OR "内脏肥胖指数"[常用字段]) AND (("糖尿病肾疾病"[常用字段] OR "糖尿病肾脏病"[常用字段] OR "糖尿病肾脏疾病"[常用字段]) OR (糖尿病肾病)) | 7 |
