# Supplementary material for: Association and predictive value of the visceral adiposity index in diabetic kidney disease: a systematic review and meta-analysis
Source: Front Endocrinol (Lausanne). 2026 Sep 9;17:1850744. doi: 10.3389/fendo.2026.1850744 (PMC13597435; doi:10.3389/fendo.2026.1850744)
Supplement: Supplementary file 3 [file Table3.docx]

Supplementary Material S3

Figure S1. Forest plot for gender subgroup analysis (OR)

Figure S2-a. Sensitivity analysis of the male subgroup

Figure S2-b. Specificity analysis of the male subgroup

Figure S2-c. Sensitivity analysis of the female subgroup

Figure S2-d. Specificity analysis of the female subgroup

FigureS3-a. Sensitivity analysis of the correlation between VAI and DKD (HR)

Figure S3-b. Funnel plot for the correlation between VAI and DKD (HR)

Figure S3-c. Begg's test for the correlation between VAI and DKD (HR)

Figure S3-d. Egger's test for the correlation between VAI and DKD (HR)

Figure S3-e. Funnel plot for the correlation between VAI and DKD (OR)

Figure S3-f. Begg's test for the correlation between VAI and DKD (OR)

Figure S3-g. Egger's test for the correlation between VAI and DKD (OR)

Figure S4-a. Sensitivity analysis of VAI for predicting DKD.

Figure S4-b. Deek's funnel plot asymmetry test for publication bias of VAI in predicting DKD

Figure S4-c. Trim-and-fill analysis of VAI for predicting DKD.

Table S1. Trim-and-fill analysis of VAI for predicting DKD

| **Stage** | **Method** | **Pooled lnDOR** | **95% CI** | | **Asymptotic** | | **Q statistic** | **df (Q)** | **No. of studies** |
| --- | --- | --- | --- | --- | --- | --- | --- | --- | --- |
|  |  |  | Lower | Upper | z_value | p_value |  |  |  |
| Before trim-and-fill | Fixed | 0.935 | 0.858 | 1.012 | 23.884 | 0.000 | 206.181 | 11 | 12 |
|  | Random | 1.437 | 1.077 | 1.797 | 7.819 | 0.000 |  |  |  |
| After trim-and-fill | Fixed | 2.173 | 2.022 | 2.336 | 21.108 | 0.000 | 389.728 | 16 | 17 |
|  | Random | 2.301 | 1.57 | 3.372 | 4.273 | 0.000 |  |  |  |

*Table Note: Trim-and-fill analysis was conducted using the linear estimator under a fixed-effects model to assess the potential impact of publication bias on the pooled diagnostic odds ratio (DOR). lnDOR, natural logarithm of DOR.
